# Game Elements in Military Trauma Care Education: Systematic Review

**DOI:** 10.2196/79163

**Published:** 2026-03-17

**Authors:** Natalia Stathakarou, Andrzej A Kononowicz, Maxine G Harjani, Cara Swain, Klas Karlgren

**Affiliations:** 1 Department of Learning, Informatics, Management and Ethics (LIME) Karolinska Institutet Stockholm Sweden; 2 Department of Bioinformatics and Telemedicine Jagiellonian University Medical College Kraków Poland; 3 Royal Centre for Defence Medicine Birmingham United Kingdom; 4 Institute of Naval Medicine Alverstoke, Gosport United Kingdom; 5 Department of Research Education and Development and Innovation Södersjukhuset Stockholm Sweden; 6 Faculty of Health and Social Sciences Department of Health and Functioning Western Norway University of Applied Sciences Bergen Norway

**Keywords:** gamification, game elements, military trauma care, simulation-based learning

## Abstract

**Background:**

Game elements may inform the design of both simulations and games. However, evidence on how individual game elements inform the design of military trauma training simulations and their educational purpose remains limited.

**Objective:**

This systematic review aimed to examine which game elements are used in the design of educational simulations for military trauma management, how they are implemented, for what purpose, and what outcomes are reported related to the game elements.

**Methods:**

This is a systematic review conducted in accordance with PRISMA (Preferred Reporting Items for Systematic Reviews and Meta-Analyses) guidelines. We included qualitative, quantitative, mixed methods, and design studies describing simulation-based training for military trauma management that incorporated game elements. Studies focusing solely on assessment, noninteractive interventions, or psychological trauma were excluded. Searches were conducted in Medline (Ovid), PubMed, IEEE Xplore, ERIC, Web of Science, ACM Digital Library, and CINAHL from inception to October 14, 2025, identifying 2487 records. Screening and data extraction were performed independently by 2 reviewers. Methodological quality was assessed using the Medical Education Research Study Quality Instrument (MERSQI) and the Côté and Turgeon grid. Results were synthesized using qualitative thematic synthesis.

**Results:**

Forty-two studies published between 1986 and 2025 were included. Most studies were conducted in the United States and included a wide range of simulation modalities and learner populations. Sixteen game elements were identified, with narrative, sensation, imposed choice, time pressure, and scoring being most prevalent. The thematic synthesis identified multiple categories describing how these game elements were implemented. Justifications for the use of game elements were rarely provided; when present, they were primarily linked to realism, emotional engagement, adaptive learning, and feedback. Elements such as badges and competition were seldom used. No study explicitly linked individual game elements to specific educational outcomes. This review is constrained by heterogeneity across studies, an imperfect fit of quality appraisal tools for some study types, and the possibility of missed studies due to search vocabulary limitations.

**Conclusions:**

This systematic review is innovative in providing the first comprehensive synthesis of how game elements are used in military trauma simulations. Unlike previous reviews, it explicitly focuses on the pedagogical purposes of these elements. It offers an overview of the prevalence of game elements in military trauma care education and synthesizes the pedagogical rationales for their use. The lack of studies explicitly linking individual game elements to learning outcomes highlights the need for more intentional research and transparent reporting. Future studies should treat gamification as a set of targeted design choices rather than as a single overarching strategy, and explore how its motivational dimensions can be effectively leveraged in military trauma training.

**International Registered Report Identifier (IRRID):**

RR2-10.2196/45969

## Introduction

Gamification is the use of game elements in an endeavor to nudge participants to perform certain actions by adopting a playful attitude [1], and it is a promising approach in health care education [2]. For example, learning activities might be designed to involve solving problems under time pressure, competing or collaborating with others, and earning points or badges [3]. Gamification has been linked to effects on motivation, behavior, and engagement, and learning [4]. In health care education, gamification and games have been shown to be at least as effective as other educational approaches, and in many studies, more effective for improving knowledge, skills, and satisfaction [2]. Gamification has the potential to improve learning outcomes, especially when it uses game elements that improve learning behaviors and attitudes towards learning [5]. Recent research indicates that gamification offers diverse and flexible strategies for enhancing clinical reasoning education across health care disciplines and settings [6]. In disaster education, gamification may enhance learners’ retention of knowledge, ability to cooperate, sense of presence, perceived realism, disaster awareness, decision-making ability, practical skills, and coping capacity [7].

Although gamification is known for its engaging and motivational benefits, its pedagogical value remains controversial [1,5]. This controversy arises because different gamification strategies may use various combinations of game elements, leading to diverse outcomes [8]. Interestingly, much of the literature treats gamification as a single approach solution, rather than acknowledging it as a collection of game elements that may vary considerably in purpose and the learning experiences that they offer.

Gamification and simulation are closely related concepts, but are often also contrasted; based on the spectrum of Qin et al [9], Ricciardi and De Paolis [10] conceptualize simulations and games as 2 extremes on a spectrum. At one end of this spectrum lie classical simulators, which are often designed for skills training and prioritize realism by replicating the real world. At the other end are games developed for fun and entertainment, often situated in entirely fictional or imaginary contexts. Between these extremes lie serious games and simulation games. Serious games are developed with nonentertainment purposes in mind, combining a high degree of realism with the entertainment elements of traditional games to facilitate skills development. In contrast, simulation games often blend imaginative or fictitious environments with simulation-based mechanics, offering engagement and the potential to support learning. Examples of serious games for military trauma training include the French Military Health Service’s 3D-SC1 game to train for and assess forward combat casualty care [11,12] and the US Army’s tactical combat casualty care simulation training program, TC3Sim [13,14].

Simulations may allow learners to experience complex situations and act as they would in a real environment. They may take several forms and provide learners with feedback. High-fidelity medical simulations are educationally effective, and simulation-based education complements medical education in patient care settings [15]. Simulation environments range from field exercises to virtual patients and virtual reality and can include a variety of different modalities in place of a human casualty, such as a manikin, a simulated patient, or a cadaver.

In military trauma care, different simulation technologies are used to train a range of technical and nontechnical competencies [16,17]. A recent scoping review [18] examined the use of simulations in military medicine and found that most studies focused on physical simulation modalities, such as manikins and task trainers, whereas only a limited number used augmented or virtual reality interventions. Simulation-based training enables the replication of austere and high-stress environments, providing a safe context for learners to practice trauma management, make decisions under pressure, and learn from errors without compromising patient safety [19]. Kubala and Warnick [20] found that knowing exactly what to expect in combat reduces fear and stress.

Military trauma care is characterized by austere environments, tactical demands, and limited medical resources that fundamentally distinguish it from civilian practice. In deployed settings, medical personnel often operate with minimal equipment and may provide care alone or under hostile conditions far from hospital facilities [21,22]. These circumstances require rapid decision-making, prioritization, and coordination under pressure, frequently with incomplete information, which increases the risk of preventable harm [23]. Military trauma also differs from civilian trauma in its organizational structures, triage systems, and treatment approaches, as well as in the nature of injuries: while civilian trauma commonly involves blunt injuries or low-velocity gunshot wounds, military injuries are often caused by blasts and high-velocity weapons [22-27]. Consequently, treatment protocols developed for civilian settings do not always translate effectively to the battlefield. Recent studies [28] emphasize the often-overlooked conditions of truly austere environments in research and education, indicating a need for trauma simulations to integrate realism and austerity. Yet, most military medical personnel receive training in civilian settings, which may not fully prepare them for managing trauma in hostile settings.

Game elements can be used in both simulations and games, serving different purposes [29]. For instance, a recent study [30] integrated game elements such as varying difficulty levels and scoring within the design of virtual patients to make them both more realistic and engaging as learning activities. Previous work analyzing game elements in education has led to frameworks and taxonomies supporting the design and evaluation of gamification in learning environments [3,31]. Extending this work, a list of game elements with the potential to support education and training in military trauma care was synthesized [30].

Incorporating game elements into simulation-based education in the field of military trauma training has the potential to increase motivation, enhance learners’ confidence, and support personalized learning [30]. Yet, literature about how individual game elements can inform the design of military trauma training simulations and influence learning outcomes is underexplored.

This systematic review aims to systematically examine the use of game elements in the design of military trauma training simulations in order to retrieve and synthesize international evidence on their design, educational purpose, and reported outcomes in trauma management training. By doing so, the systematic review seeks to inform simulation design practices and identify directions for future research. The research questions guiding this systematic review are:

1. What game elements are used in the design of educational simulations in the context of military trauma management?
2. How are the identified game elements used?
3. What is the purpose of using game elements in the design of educational simulations in military trauma management?
4. What outcomes are reported related to the game elements?

## Methods

### Protocol and Registration

A systematic review protocol for this study has been published in JMIR Research Protocols (PMID: 37682596) [32]. No changes were made to the planned synthesis methods after protocol publication. The results are reported in accordance with the PRISMA (Preferred Reporting Items for Systematic Reviews and Meta-Analyses) guidelines [33]. The PRISMA checklist, the PRISMA-S checklist, the PRISMA for Abstracts checklist, and SWiM (Synthesis Without Meta-analysis) reporting items are provided in Multimedia Appendices 1-4, respectively.

### Inclusion and Exclusion Criteria

We included both qualitative and quantitative empirical and design studies that addressed different types of simulation interventions, which incorporated game elements. Game elements were identified using published gamification frameworks in a deductive-inductive manner [3,30,31]. We included studies that incorporated game elements designed to enhance the education and training of military trauma management. The included simulation interventions had a clear educational purpose within this context. In this systematic review, the included gamified simulations had some degree of interactivity, allowing the scenarios to unfold in response to the learners’ actions. Studies in which participants passively observed a scenario (eg, a video clip) were excluded. Studies using interventions primarily for patient education, rehabilitation, teleconference, treatment, and decision-support were considered outside the scope of this review. We excluded studies where the training only focused on individual body parts. We also excluded studies in which gamification was used solely for assessment, unless the assessment served an educational purpose. We included studies focusing on military trauma management and excluded those addressing psychological trauma, because it belongs to a different educational context and learner group. We included studies that had a link to military medicine, even if the learner population that received the simulation was linked to a civilian context. Game elements only taking place outside the simulation, such as scoring conducted by instructors who observed or assessed participants after a simulation session, were also excluded. Several simulations incorporated teams to develop team-relevant competencies, and a “team” is recognized as a game element in the Maheu-Cadotte framework [3]. We only included studies that introduced teamwork with a gamification purpose and excluded studies where teamwork was present merely because it was inherently a part of the simulation, such as when practicing communication skills.

### Search Methods for Identification of Studies

We explored the databases/search engines: Medline (Ovid), PubMed, IEEE Xplore, ERIC, Web of Science, ACM Digital Library, and CINAHL with the help of librarians at the University Library of Karolinska Institutet. We included all articles regardless of publication language. All articles published up to October 14, 2025, were retrieved. With the help of the librarians, we conducted a search of citations and references in CitationChaser. Each database was searched independently, and no multi-database search function was used. No limits or filters were applied. The search strategies did not undergo a formal peer review, although they were developed by 2 experienced librarians and iteratively tested across several rounds. No study registries were searched. No additional online or print sources were purposefully browsed (eg, tables of contents, conference proceedings, or websites) beyond the citation and reference searching described above. Search strategies were developed specifically for this review in collaboration with librarians and were not adapted or reused from previous literature reviews. The search strategy is included in Multimedia Appendix 5 [34].

### Selection of Studies

We imported all identified references to the Rayyan open-source web system [35]. Full-text versions of the included abstracts were retrieved via the university library. We did not contact authors or other stakeholders to obtain additional studies or data. Two researchers independently assessed the identified studies based on the inclusion and exclusion criteria. Any disagreements were resolved through discussion between the 2 reviewers. If no agreement could be reached, a third researcher was consulted. The selection process was represented using a PRISMA flow diagram [33], including the actual number of studies included and excluded at each stage of screening.

### Data Extraction and Management

The data extraction sheet and initial coding frame for identifying game elements and outcomes were predefined and published in the registered review protocol [32]. To ensure consistency and a shared understanding of the coding approach, all authors jointly piloted the extraction process on 3 studies during the protocol development stage. This pilot served as a coder calibration exercise to refine code definitions and decision rules.

Throughout the review, the first author was paired with each coauthor during data extraction and coding to maintain alignment and consistency in interpretation. Two researchers independently extracted and managed the data for each included study using the finalized structured form. Any discrepancies were discussed until consensus was reached, and a third author was consulted when necessary.

### Data Analysis, Synthesis, and Reporting

A qualitative data analysis combined with thematic synthesis [36] was conducted to answer the research questions. Data synthesis was performed using structured Excel spreadsheets; no qualitative analysis software was used. The simulations described in the included studies were classified according to categories proposed in earlier literature [16,17]. Virtual patients were categorized following the classification framework introduced by Kononowicz et al [37]. To answer the first research question, the reviewers compared extracted data to ensure consistency in coding and interpretation of game elements across studies. The data extraction process was guided by a predefined list of game elements based on existing frameworks [3,30,31].

To address the second research question, we synthesized the data further by grouping realizations of each game element. This involved reviewing how each element was operationalized across studies and clustering similar patterns into subcategories. This synthesis allowed us to develop categories that describe the implementation of each game element. The process was iterative and collaborative, followed by frequent discussion between all authors to refine the categories, ensure consistency, resolve discrepancies, and reach a consensus.

To answer the third research question, a thematic synthesis approach was applied. Paired researchers worked independently to review each study and extract passages of text that provided a rationale, justification, or implied educational purpose for the use of specific game elements. These included both explicit explanations and implicit meanings inferred from the study context. Subsequently, we clustered similar meanings into broader themes that captured the underlying educational or experiential intentions behind the use of the identified game elements. This involved a process of thematic grouping that connected the extracted rationales with the corresponding game elements identified earlier. The mapping was conducted by 2 researchers and discussed by all authors. Themes were constructed inductively from the data. Each theme was then linked to one or more game elements.

To address the fourth research question, we collected information on the reported educational or performance outcomes of the simulations attributed to individual game elements as described in each study. Data extraction focused on outcomes related to knowledge, skills, and attitudes, specifically engagement, confidence, and secondary outcomes, like accessibility and cost-effectiveness. Although outcomes were recorded alongside the included studies, no formal synthesis was conducted in the located studies to systematically link specific game elements to particular outcomes. This was due to limitations in the way outcomes were reported across studies as a combined effect of all game elements, rather than separately for each game element, and hence the lack of explicit connections between individual game elements and measured results.

No standardized outcome metrics or effect-size transformations were applied. Given heterogeneous outcome reporting and the absence of comparable effect estimates, findings were synthesized using qualitative thematic synthesis, focusing on the implementation and pedagogical purpose of game elements. All included studies contributed to the synthesis; no studies were selected or excluded from the synthesis based on study design or quality assessment, which was used descriptively to support interpretation rather than to weight or filter findings.

Findings were presented using structured tables and narrative summaries. Tables reported key study characteristics (eg, study design, simulation type, learner population) and the presence and implementation of individual game elements; studies were organized descriptively rather than ordered by effect size or risk of bias, as no comparable effect estimates were available.

### Quality Appraisal

The quality of the included studies was assessed using 2 established instruments. For quantitative and mixed methods studies, we applied the Medical Education Research Study Quality Instrument (MERSQI) [38]. For research that reported results obtained exclusively through qualitative methods, such as interviews or focus groups, we used the quality appraisal grid developed by Côté and Turgeon [39]. Studies focusing solely on the design and development of simulations were not included in these quality assessments as they fall outside the intended scope of both MERSQI and the Côté and Turgeon grid [39]. Methodological quality was appraised descriptively to support interpretation of the evidence base. Certainty of the synthesis findings was not formally assessed using a grading framework.

### Ethical Considerations

This systematic review does not involve processing of sensitive personal data and therefore ethical approval is not required according to the Swedish Ethical Review Act.

## Results

### Included Studies

**Figure 1:**
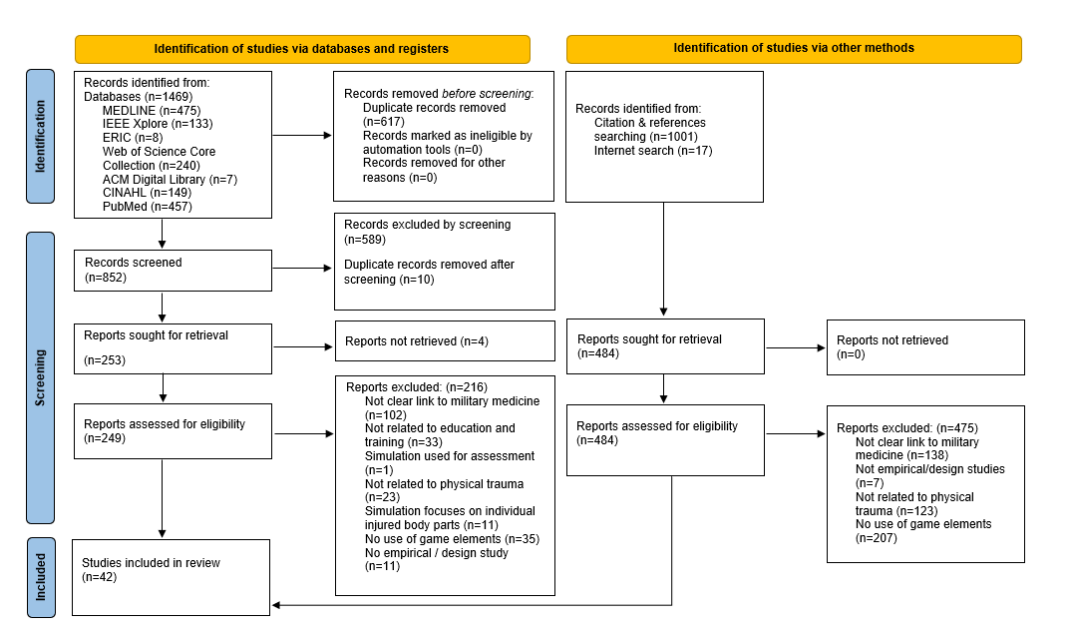
PRISMA (Preferred Reporting Items for Systematic Reviews and Meta-Analyses) diagram.

In total, 42 studies were included. Figure 1 presents the PRISMA flowchart showcasing the search and inclusion process. If an article was excluded for multiple reasons, only the first applicable exclusion criterion, as defined in the published review protocol [32], was recorded.

### Article and Study Characteristics

The studies covered a variety of simulation types used for training purposes, including nondigital games, virtual patients, virtual patient games, live simulations, standardized patients, mannequins, and virtual and mixed realities. Most of the studies were conducted in the United States, followed by studies originating from Europe. The learner populations involved were diverse, ranging from hospital decision-makers, surgeons, soldiers, and combat medics to medical students and emergency response teams. Table 1 provides information about the general characteristics of the studies included, specifically the type of simulation, the country in which the study was conducted, and the learner population.

**Table 1:** Included studies, type of simulation, and learner population.

Lead author and year	Simulation type	Learner population	Country
Achatz et al (2020) [40]	Nondigital game/board game	Hospital decision makers	Germany
Arora et al (2014) [41]	Human Standardized Patient	Surgeons, anesthesiologists, physicians, and nurses	United Kingdom
Badler et al (1996) [42]	Virtual patient game	Combat Medics	United States
Baird et al (2020) [43]	Virtual patient - High Fidelity Software Simulation	Thermal injury treatment providers	United States
Beaven et al (2021) [44]	Cadaver and High Fidelity Manikins	National Guard members, doctors, nurses, physicians, operating department practitioners, medical students	United Kingdom
Brown et al (2016) [45]	Virtual and mixed realities	Combat Medics	United States
Chi et al (1996) [46]	Virtual patient game	Combat Medics	United States
Chi et al (1997) [47]	Virtual patient game	Combat Medics	United States
Cohen et al (2013) [48]	Virtual patient game	Clinicians practicing trauma leadership; clinical major incident coordinator/silver commander.	United Kingdom
Couperus et al (2019) [49]	Virtual and mixed realities	Emergency military physicians	United States
Couperus et al (2020) [50]	Virtual and mixed realities	Emergency military physicians	United States
DeFalco et al (2017) [13]	Virtual patient game	Combat medics	United States
de Lesquen et al (2022) [51]	Virtual patient game	Emergency doctor	France
de Lesquen et al (2023) [52]	Virtual patient game	Prehospital physicians	France
Du et al (2022) [53]	Virtual and mixed realities	Military medical students	China
Freeman et al (2001) [54]	Virtual and mixed realities	Navy medical providers (paramedics)	United States
Goolsby et al (2014) [55]	Immersive virtual environment	Military medical students	United States
Hemman (2005) [56]	Virtual patient - High fidelity software simulation	Combat medics	United States
Henderson et al (1986) [57]	Virtual patient - High fidelity software simulation	Medical students	United States
Henderson et al (2020) [58]	Virtual patient game	Combat medics	United States
Kyle et al (2004) [59]	High fidelity Manikins and Human standardized patient	Physicians, nurses, paramedics, professional scientists, military officers, lawyers, career politicians, consultants from nongovernmental organizations, administrators, intelligence officers, and logistic personnel.	United States
Lombardo et al (2022) [60]	Virtual and mixed realities	Emergency medicine residents, attendings, medical students, physician assistants, army medics, and nurses	United States
Lu et al (2023) [61]	Virtual and mixed realities	Combat medics and military surgeons	China
Lennquist Montán et al (2014) [62]	A card game in a live exercise	Physicians, nurses, ambulance/paramedics, Military staff, administrators, also collaborating agencies (rescue services, the police)	Sweden
Netzer et al (2015) [63]	High fidelity manikins	Navy Emergency Medical Teams, military physicians	Israel
Pasquier et al (2016) [11]	Virtual patient game	Soldiers, combat medics	France
Planchon et al (2018) [12]	Virtual patient game	Soldiers, combat medics	France
Qin et al (2024) [64]	Virtual and mixed realities	Combat medics, nurses	Israel
Rabotin et al (2023) [65]	Virtual and mixed realities	Paramedics, physicians	Israel
Satava and Jones (1996) [66]	Virtual and mixed realities and wearables	Combat medics	United States
Sonesson et al (2023) [67]	Interactive patient scenarios	Military trauma teams	Sweden
Sotomayor (2008) [68]	Virtual patient game	Combat medics	United States
Sotomayor (2010) [14]	Virtual patient game	Combat medics	United States
Stansfield et al (1998) [69]	Virtual and mixed realities	Combat medics	United States
Stathakarou et al (2024) [30]	Interactive patient scenarios	Combat medics	Sweden
Stone (2005) [70]	Virtual patient game	Military trauma surgeons	United Kingdom
Stone (2011) [71]	Virtual patient game	Military trauma surgeons	United Kingdom
Stone et al (2017) [72]	Virtual and mixed realities	Medical Emergency Response Teams (MERTs)	United Kingdom
Tretyak et al (2025) [73]	Virtual and mixed realities	Medical personnel and trainees involved in tactical emergency or combat casualty care	Austria
Wier et al (2017) [74]	Immersive virtual environment	Medical Emergency Response Teams (MERTs: physicians, nurses, medics)	United States
Willy et al (1998) [75]	Virtual patient - High fidelity software simulation	Military physicians	Germany
Zhu et al (2024) [76]	Virtual patient game	Mobile medical logistics teams: background in medicine, nursing, logistics	China

### Game Elements Identified in the Design of Military Trauma Simulations

**Figure 2:**
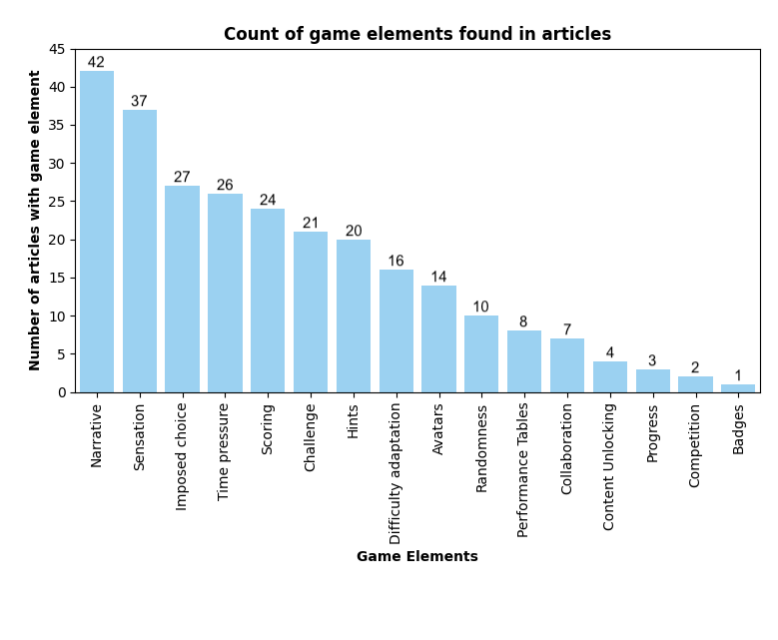
Synthesis of game elements identified in the design of military trauma simulations.

Figure 2 presents a synthesis of the 16 game elements identified in the design of military trauma simulations across the included studies. Multimedia Appendix 6 [11-14,30,40-76] provides a detailed categorization of the game elements identified in each of the 42 studies. The definitions of the game elements were derived from previous literature [3,30,31]; narrative refers to the structured sequence of events and decisions shaping the learner’s experience, while sensation captures the use of visual or auditory stimuli to enhance immersion. Imposed choice describes situations in which learners must select one option to progress, whereas time pressure requires actions or decisions within urgency or a limited timeframe. Scoring provides quantitative feedback with points, and hints refer to clues that support learners without revealing the correct answer outright. Challenge encompasses mechanisms designed to test abilities, and difficulty adaptation refers to the dynamic complexity of tasks and adjusting. Avatars function as digital representations of learners or patients, and randomness introduces unpredictable aspects in the scenario. Performance tables present detailed summaries of accomplishments across tasks, and collaboration encourages learners to work together toward shared objectives. Content unlocking restricts access to new material until specific criteria are met, progression visualizes the learner’s development over time, competition supports comparisons with mechanisms such as leaderboards, and badges serve as symbolic markers of achievement for completing tasks. The definitions of the game elements are summarized in Multimedia Appendix 7.

### Application of Game Elements in Military Trauma Simulations

Table 2 provides a thematic synthesis of how game elements were used in the included studies, outlining different categories of how game elements can be used to inform educational simulations in trauma care.

**Table 2:** Thematic synthesis of how the game elements were used in the included studies.

Game element	Application of game elements in military trauma simulations
Narrative	An interactive scenario that unfolds on the basis of the learners’ decisions [11-14,30,40-76]
Sensation	High environmental fidelity by replicating battlefield conditions [41,44,59] Virtual fidelity in the digital environment to depict austerity, such as realistic portrayals of injury or digitally recreated battlefield environments [11-14,30,42,43,45-54,57,60-62,64,66,68-71,73,75,76] Mixed reality and interaction with objects in both physical and virtual environments [55,65,72,74]
Imposed choice	Presentation of explicit decision options on patient cards [40] Digital menu with predefined decisions [11-13,30,43,45-48,50,52,54,57,58,60,61,64,65,67-71,75,76]
Time pressure	Scenarios incorporating explicit time constraints [11,40,70-72,74] Tasks requiring immediate action to simulate urgency [30,48,50-52,55,59,73] Display of visible timers or auditory cues such as ticking clocks to reinforce urgency [45,51,54,57,64,75,76] Delayed decisions negatively affecting patient outcomes, potentially leading to patient death [13,48,50,62,69-71] Timely decision-making contributing positively to performance metrics [42,65]
Scoring	Quantifying learner performance based on patient health outcomes, such as trauma scores or health points [40,42,46,47,62,69] Final scoring mechanisms summarizing overall performance or decision quality at the end of the scenario [11-13,30,41,45,51,52,60,61,63,65,70,76] Real-Time Scoring: Continuous assessment during gameplay [30,40,45,49,50,64] Penalty-Based Scoring: Deducting points for incorrect decisions [65] Competitive scoring formats allowing ranking or comparison between participants [57] Scoring linked to resource management, rewarding efficient allocation [57]
Hints	Human instructor–guided feedback during simulation sessions [42,44,55,59] Computerized real-time audio feedback [45] Simulated patient cues through physiological responses, appearance, or facial expressions [13,42,48-50,64,66,69,70,75] Computerized feedback via virtual patient interactions or dialogue boxes [30,56,57,68,76] Virtual instructor or colleague intervening when poor decisions occur [11,57] Feedback on inappropriate or excessive resource use [57] Contextual hints through simulated live TV news updates in real time [59]
Challenge	Managing complications and unforeseen events [40,43,48,63] Exposure to distracting sounds or visual elements [44,45,72,73] Performing difficult triage decisions, prioritizing treatable patients over those unlikely to survive [61] Decision-making under conflicting or incomplete information [40,59] Identifying concealed or initially nonobvious injuries [30,66] Providing care in austere or unfamiliar environments [30,41,42,44,63,66,69,72,73] Encountering unwinnable cases with inevitable failure outcomes [13,30,58] Experiencing learner death when safety precautions are neglected [30,68]
Difficulty adaptation	The “game master” can adjust the scenario’s difficulty based on participant performance or situational needs [40,62,63] Learners are able to select the initial difficulty level before the scenario [11,45] Gradually increasing or varying levels of challenge [11,13,30,45,50,51,54,57,58,67,75] Dynamic difficulty in the scenario based on participants’ performance [42,63,65]
Avatar	Patient cards representing virtual patients [40] Learner avatars enabling player interaction through a virtual self or character [42,43,48,54] Customizable learner avatars [45] Avatars mirroring learners’ physical movements in real time [46,47,66,69] Patient avatars representing casualties within the simulation [51-53,73]
Randomness	Unexpected resource problems: missing equipment, availability of staff, hospital resources [40,62] Scenario variability in incident type, number of casualties, environmental austerity, weather conditions, and patient injuries or physiology [42,43,45,50-52,62,64] Identical treatments do not always produce the same outcomes [57]
Performance tables	User performance summaries comparing actions against predefined “gold standard” treatment procedures [45] Event timing and adherence to trauma resuscitation protocols [65] Interactive display of casualty vital signs and key events with a complete performance log [45] Performance grids integrating scoring and structured debriefing [11,51,52,64,70,76]
Collaboration	Multi-avatar collaboration between human players within the same simulation environment [42,45] Collaborative tasks designed to enhance understanding of different professional roles [45,53] Collaboration with virtual or artificial intelligence–driven team members and avatars [50,53,57,60,72]
Content unlocking	Completion of required treatment steps to progress within the scenario [45] Selection of specific choices or actions to advance gameplay [30,40] Demonstration of proficiency or task mastery to unlock subsequent levels [11]
Progress	Sequential management and triage flow of patients throughout the scenario [40] Grid-based visualization of patient health and status [51] Rescue progress bar displaying ongoing operations and cumulative training score in real time [76]
Competition	Scores used to compare and rank learners, enabling performance competition among peers [11,57]
Badges	Bronze, silver, and gold medal graduation according to the scoring system, integrating time and actions delivered [11]

### Purposes of Using Game Elements in the Design of Simulation in the Included Studies

While most of the studies did not attempt to attribute the use of game elements to a specific educational purpose, the following studies were identified that justified the use of specific game elements. Table 3 summarizes the 9 identified themes explaining why specific game elements were integrated into the simulation design. Supporting data excerpts from the included studies are provided in Multimedia Appendix 8 [11,13,30,40,41,43,45-47,52-54,57,60,62,63,65,69,73-75].

**Table 3:** Justification themes of using game elements in the included studies.

Theme	Game element
Realism and emotional engagement	Narrative [30,41,43,45,51,57,63]; Sensation [11,30,41,43,45,46,51,57,63]; Time pressure [30,40,73]; Avatar [45,51]; Randomness [45,57]; Challenge [45,73]; Imposed choice [30,47,62,75]; Collaboration [53]
Adaptive learning and feedback	Difficulty adaptation [45,51,60,65]; Scoring [11,47,51,60]; Performance tables [11,45,51]
Affective learning	Challenges [12]
Learner agency	Imposed choice [12]
Challenge the learners	Difficulty adaptation [54]
Risk-free experiential learning	Narrative and Sensation [54], Time pressure [69]
Motivation and engagement	Competition and Scoring [11]
Situational awareness	Challenge [69]
Emotional regulation and overcoming anxiety	Challenge and Sensation [74]

### Reported Outcomes Associated With Game Elements

None of the included studies correlated individual game elements with the reported outcomes. Instead, the effects on knowledge, skills, and attitudes were attributed to the impact of the simulations as a whole.

### Quality Appraisal of Studies

We evaluated 22 studies with MERSQI with scores ranging from 5 to 15.5 and a median score of 9.5 (Table 4). The most common methodological limitations identified included reliance on a single cohort without comparison groups, the use of nonvalidated evaluation instruments, and a focus on outcomes such as satisfaction or basic knowledge and skills acquisition. Since our inclusion criteria did not restrict studies to pre-post designs, the MERSQI tool items were not applicable in all cases, which may have contributed to lower scores in some studies. We evaluated only 2 studies with the grid by Côté and Turgeon [39] that used qualitative data collection methods. We excluded 18 design and development studies from the quality appraisal due to their descriptive nature, which falls outside the scope of both MERSQI and the Luc Côté grid. The evaluation score of each study is briefly available in Tables 4 and 5. Design studies were not assessed with formal quality appraisal tools [11,42,43,45-47,49-51,54,57,58,66,69-72,75]. Table 5 includes the 2 studies that were appraised using the Côté and Turgeon grid [39].

**Table 4:** Quality appraisal for studies using the Medical Education Research Study Quality Instrument.

Study	Study Design (max=3)	Sampling (max=3)	Data type (max=3)	Validity (max=3)	Data analysis (max=3)	Outcomes (max=3)	Score (max=18)
Achatz et al (2020) [40]	1	1.5	1	0	2	1	6.5
Arora et al (2014) [41]	1	2	1	1	3	1.5	9.5
Beaven et al (2021) [44]	1.5	2	3	1	3	1.5	12
Cohen et al (2013) [48]	1	0.5	3	2	3	1.5	11
DeFalco et al (2017) [13]	3	1	1	1	3	1.5	10.5
de Lesquen et al (2023) [52]	2	2	3	1	3	1.5	12.5
Du et al (2022) [53]	3	2	1	0	2	1.5	9.5
Goolsby et al (2014) [55]	1.5	2	1	0	2	1	7.5
Hemman (2005) [56]	1.5	2	1	0	3	1.5	8.5
Kyle e al (2004) [59]	1	0	1	0	2	1	5
Lombardo et al (2022) [60]	1	0.5	1	0	2	1	5.5
Lu et al (2023) [61]	1	1.5	1	0	2	1	6.5
Lennquist Montán et al (2014) [62]	1	3	1	0	2	1	8
Netzer et al (2015) [63]	1.5	2	3	2	3	1.5	13
Planchon et al (2018) [12]	3	2	3	2	3	1.5	14.5
Qin et al (2024) [64]	1.5	0.5	3	0	3	1.5	9.5
Rabotin et al (2023) [65]	1	0.5	1	0	2	1	5.5
Sonesson et al (2023) [67]	2	0.5	1	0	2	1.5	7
Sotomayor (2008) [68]	3	2	3	2	3	1.5	14.5
Sotomayor (2010) [14]	3	0.5	3	1	3	1.5	12
Wier et al (2017) [74]	2	2	3	3	3	1.5	14.5
Zhu et al (2024) [76]	3	2	3	3	3	1.5	15.5

**Table 5:** Quality appraisal for studies using the Côté and Turgeon grid [39].

Study	Introduction (max=2)	Methods (max=5)	Results (max=2)	Discussion (max=2)	Conclusion (max=1)	Total (max=12)
Stathakarou et al (2024) [30]	2	5	2	2	1	12
Tretyak et al (2025) [73]	2	4	2	2	1	11

## Discussion

### Principal Findings

In this systematic review, we investigated the use of game elements in simulations for military trauma management. The results provide insights into the common application of the game elements and summarize the justification for their use. However, no study explicitly linked individual game elements to specific learning or performance outcomes.

In health care education, gamification and games have been shown to be at least as effective as other educational approaches, and in many studies, more effective for improving knowledge, skills, and satisfaction [62]. Gamification has the potential to improve learning outcomes, especially when it uses game elements that improve learning behaviors and attitudes towards learning [63]. However, many of the studies included in previous reviews are of low quality, lack a sufficient focus on specific game elements [62], and have methodological limitations [5].

Although games and simulations have been used in military training for centuries [77], research on how individual game elements can inform the design of military trauma training simulations and influence learning outcomes is underexplored. The question of linking specific game elements to outcomes has been raised in gamification studies [8,78] but received little attention in military trauma contexts. Even if the link between game elements and learning outcomes were established, designers must account for the fact that individual elements can be implemented in multiple ways, leading to variation in results.

Clarification studies, which explore how underlying mechanisms account for the observed effects [79], are uncommon in gamified learning research [5] and are largely absent in military trauma contexts. Such studies could provide insights into the mechanisms involved in gamified learning, inform the design of gamified learning approaches [4,5], and contribute to the understanding of the relationship between design principles and learning outcomes, such as clinical reasoning [80].

As early as 1957, Caillois [81] described characteristics that distinguish games from other forms of activity, including fun, uncertainty, detachment from real life, nonproductivity, the presence of specific rules, and fictitious settings. Garris et al [82] proposed 6 dimensions that distinguish games from traditional simulations, including fantasy, mystery, sensory stimuli, rules and goals, controls, and challenge.

In this systematic review, narrative and sensation, as the most frequently used game elements in the reviewed simulations, contribute to the fictitious settings by fostering immersion and enabling environmental recreation. Narrative was frequently combined with sensation, the use of sensory stimuli, such as visual and auditory cues, to enhance immersion, often reflecting the austerity and constraints of military trauma contexts. In the application of digital technology, multimedia has been shown to enhance learning outcomes such as satisfaction, achievement, motivation, and attention [83]. In live simulation exercises, sensation was often conveyed by replicating battlefield conditions. This was achieved by conducting simulations outside the classroom or in rare environments, such as simulated deployed hospitals or ships, and by incorporating battlefield sounds and stress-inducing elements to recreate austerity [41,44,59].

Difficulty adaptation was identified in 16/42 included studies. The level of difficulty is a critical factor in learning; it should remain below the maximum capacity of learners’ knowledge to maintain motivation and facilitate effective learning. When difficulty exceeds learners’ capabilities, performance and engagement may decrease [84]. In the included studies, difficulty adaptation was implemented in various ways, for instance, in a classroom, with an instructor adjusting the level to the learners’ performance [40,62,63] or by allowing the learners to select different levels prior to the start of the game [11,45].

“Challenge” as a game element was identified in several studies. This differs from the level of difficulty, which corresponds to the level of knowledge and the skills the learners need to apply to perform the task, as challenge often involves dealing with complications and unforeseen circumstances [40,43,48,63]. Examples include the requirement to make difficult triage decisions (eg, prioritizing treatable patients over those unlikely to survive) [61], decision-making with conflicting information [40,59], distractors such as background noise and visual disturbances [44,45,72,73], or emotionally charged scenarios and unwinnable cases [13,30,58].

Simulations also frequently included imposed choice, which presented users with predefined options or decision menus. Additionally, scoring and time pressure were commonly used to provide feedback or assess learner performance and simulate the urgency of trauma care, respectively. Scoring was included in the total of 24/42 studies. In several cases, it was based on the performance of the decision-making of the learners, providing learners with performance-based feedback [11-13,30,41,45,51,52,60,61,63,65,70,76]. A recently published study [85] proposes that learners should be given enough opportunities to fail and try again to support serious gaming for disaster preparedness; however, without direct feedback after failure. While repetitive training enables learners to refine their problem-solving strategies over time, a principle in line with Ericsson et al’s [86] theory of deliberate practice, feedback combined with scoring may enable reflection in military medicine [30].

In total, 26/42 studies included time pressure, which was introduced both directly and indirectly within several simulations. For example, in some cases, time pressure was made explicit through the display of a ticking clock on the screen [45,51,54,57,64,75,76], whereas in others it was conveyed more implicitly by prompting learners to make rapid decisions under conditions where delays could negatively affect patient outcomes and performance scores.

While some of the game elements, such as narrative, sensation, and time-pressure that simulate contextual austerity of the military environment were often present, other types of game elements that are commonly associated with the playful and entertaining side of gamification were underrepresented. For example, badges appeared only in the form of medals in Pasquier et al [11]. Games have a long history in military medical training, yet the emphasis has perhaps traditionally been placed on their potential to mimic an austere environment, rather than on entertainment. It is possible that the playful dimensions of gamification were intentionally de-emphasized to maintain the seriousness of military trauma training. Alternatively, such elements may have been included in the simulations but not explicitly described in the study reports.

Competition was identified only in 2 studies [11,57], and in both cases, it was accompanied by scoring. Competition has been widely discussed in educational theory for its potential to enhance motivation and engagement. Malone and Lepper [87] emphasized competition as a mechanism that fosters intrinsic motivation by providing learners with clear goals and immediate feedback, often driving individuals to achieve better outcomes when compared with others. Van Eck and Dempsey [84] extended this idea, suggesting that competition may stimulate both intrinsic and extrinsic motivation: “For learners who are extrinsically motivated by social standing and recognition, competition against other individuals may serve to increase their efforts and perseverance in the instructional game to gain standing among their peers. Learners who are intrinsically motivated may likewise compete against their own score to see how much better they can do” [84]. In the same study, competition is linked to the concept of challenge.

Johnson and Johnson [88] broadened the concept by emphasizing collaborative competition, where individuals or teams compete in a way that supports group cohesion and shared learning objectives. This form of competition minimizes the potential negative effects, such as stress or discouragement. Collaboration was discussed in studies as training team skills for trauma training was the purpose of the intervention. In this review, however, we only included collaboration as a game element when it extended beyond the general goal of team training and was implemented in a distinct or designed manner. Examples included multi-avatar collaboration between human players within the same simulation environment [42,45], collaborative tasks designed to enhance understanding of different professional roles [45,53], or collaboration with virtual or artificial intelligence–driven team members and avatars [50,53,57,60,72].

Understanding the motivations behind the use of game elements is challenging, particularly in studies where design intentions are not made explicit and game elements are not formally acknowledged. In the studies where an explicit rationale was provided, linking game elements to justifications of their purpose resulted in the identification of 9 themes: realism and emotional engagement [11,30,40,41,43,45-47,51,53,57,62,63,73,75], adaptive learning and feedback [11,45,47,51,60,65], affective learning [12], learner agency [12], challenge the learners [54], risk-free experiential learning [54,69], motivation and engagement [11], situational awareness [69], emotional regulation and overcoming anxiety [74].

When examining the stated purpose behind the use of game elements, we found that elements such as narrative and sensation were most frequently justified in terms of their contribution to realism and immersion. In contrast, most other game elements were either not discussed at all or not explicitly linked to any specific educational function. Interestingly, even in studies that mentioned motivational aims in their background sections, game elements were rarely linked to motivation or engagement. For instance, the study by Achatz et al [40], which used a broad range of game elements mentioned in the background: “The didactic approach and the course structure ensure the interest, motivation, and progress of the target group, namely experienced clinical decision makers.” Despite this statement, looking into the use of game elements and their justification, time pressure was not presented as a motivational design choice but rather as a mechanism to simulate real-time conditions. We only identified one study [11] linking competition and scoring to motivation and engagement: “Furthermore, through the processes of scoring and gamification applied in 3D-SC1, the trainee is motivated to improve his personal experience. He also shares his scores with his peers in a competitive and engaging challenge”.

When examining the stated purpose of the simulation itself in a broader way rather than looking at the scope of specific individual game elements, we found that in the few studies explicitly referring to gamified simulations or serious games, the primary goals were often to attract and sustain learners’ interest, and to enhance motivation and engagement. For instance, Planchon et al [12] emphasized the motivational and entertaining aspects of serious games, noting: “SGs are similar to video games in that they are engaging, rewarding, and fun. However, at the same time, they can also be used to educate or train.” In contrast, studies that did not explicitly use the terms game or gamification tended to incorporate game elements to enhance realism and immersion, rather than playfulness or entertainment. These elements were often integrated to replicate the conditions and constraints of real-world environments. For example, Beaven et al [44] noted: “Our aim was to deliver a highly realistic, immersive simulation training experience, teaching both technical and nontechnical skills necessary for the management of war injuries in the austere environment of a far forward surgical facility. Recreating the physical and psychological work environment in a realistic way was desirable in order to encourage people to behave as they would in real life. By fostering this real-life behavior, the participants are better able to imagine the authentic scenario, and training becomes more immersive as a result.” In such cases, game elements appeared to function to enhance realism and authenticity rather than as mechanisms to directly foster learner motivation or engagement. Other studies, such as Sotomayor [14], noted that games: “appeal to the younger generation that has been exposed to their use since early age. Motivation is a big factor observed within the training audience”. However, even in such cases, the justification remained general and did not extend to a discussion of how specific game elements serve defined educational purposes and support motivation.

Realism in simulation encompasses physical, conceptual, and emotional fidelity, each contributing to the authenticity of the learning experience [89]. Advances in artificial intelligence have further expanded the potential for realism by enabling more dynamic and immersive environments [30,90]. On the other hand, some research points out a cognitive bias towards highly realistic and technically advanced learning tools; this review included 2 studies [70,71] which noted that while multimedia special effects may appeal to avid gamers, they can also distract serious users and impair performance. Effective multimedia materials require careful attention not only to managing cognitive load, but also to ensuring that the chosen media formats support, rather than hinder, learning [91].

None of the included studies explicitly linked individual game elements to reported learning outcomes. When the outcomes of the simulations were considered more broadly, most studies described positive educational effects. This observation aligns with findings from a recent systematic review on gamification in disaster education [92], which concluded that gamification could enhance competencies such as emergency response, decision-making, and teamwork in disaster nursing education, and can support learning engagement through game elements such as cooperation, competition, scoring, and scenario-based activities. However, that review also did not analyze the direct relationship between specific game elements and particular learning outcomes.

### Design Implications

The findings of this systematic review suggest that game elements should be treated as targeted design choices that are selected to serve a clearly stated pedagogical intention, rather than added as generic “gamification.” A practical starting point is to define how a simulation is intended to be supported through game elements, and then to implement the corresponding elements as a coherent configuration. For simulations aiming to strengthen realism and emotional engagement in austere trauma contexts, narrative and sensation could be used to create a realistic and immersive experience, and then intentionally reinforced through time pressure, imposed choice, and selected forms of challenge, randomness, avatar use, or collaboration to recreate uncertainty, constraints, and team demands that characterize deployed care. These game elements were previously identified to contribute to realistic tactical experiences for civilian and military trauma care [30] and align with the game elements reported in Table 3.

When the intention is adaptive learning and feedback, limited evidence from this review suggests the use of game elements such as difficulty adaptation, scoring, and performance tables. In the studies included in this review, motivation and engagement were rarely justified at the game element level and, when they were, this was linked to competition and scoring [11]. However, when deciding on what game elements to use, educators and designers might want to consider that time pressure in simulation might not correspond to the time required in the field, and some of the elements, such as scoring and competition, might be perceived as misaligned with the learning goals [93]. Therefore, a good practice might be to present such elements as optional and configurable features [93].

Finally, because none of the included studies linked individual game elements to outcomes, educators, designers, and researchers are invited to consider the hypothesized mechanism associated with each chosen element and to align evaluation with those mechanisms. In practice, this means moving beyond whole-simulation outcome claims and using designs and measures that can test element-level contributions, thereby improving understanding of how specific elements shape decision-making and the learning experience.

### Methodological Considerations and Limitations

This review was designed and conducted as a systematic literature review, following a published protocol [32] and transparent, reproducible procedures for searching, screening, extraction, synthesis, and quality appraisal [94]. The purpose was to retrieve international evidence of the impact of game elements in trauma management training and inform design practices and future research. Although the first research questions involved descriptive accounting of design features, which might be considered a hallmark of a scoping review [94,95], the subsequent thematic syntheses produced a structured and critically appraised summary of the use and educational justification for game elements in trauma management simulation reported in the literature. However, we acknowledge that the borderlines between different types of systematic reviews are often blurred in practice [94-96].

A striking finding was that none of the included studies correlated individual game elements with the reported outcomes. Instead, outcomes were generally attributed to the simulation as a whole. Consequently, evidence cannot be synthesized quantitatively on the level of specific game elements’ effectiveness. This should be interpreted as a finding about the state of the literature, rather than a limitation of the systematic review methodology.

When initiating this review, the original intention was to identify studies that explicitly addressed gamification in the context of military trauma training. Acknowledging that the literature on this topic may be sparse, the research questions were adapted to instead explore the use of game elements in the design of simulations in this context. This approach was based on the understanding that even in the absence of an explicitly acknowledged gamification strategy, game elements may still be purposefully or unintentionally embedded in the instructional design of simulations, potentially influencing the learning outcomes. As anticipated, direct comparisons between gamified and nongamified conditions or otherwise isolated reported effects of specific game elements were not observed.

We conducted a quality assessment of the included quantitative, qualitative, and mixed methods studies using 2 established tools: the MERSQI [38] and the Côté and Turgeon grid [39]. Design and development studies were not assessed using these instruments because they fall outside their intended scope. The appraisal was used descriptively to characterize methodological features and limitations across the included studies and to support interpretation of the evidence base, rather than to weight the thematic synthesis. This approach is consistent with review typologies describing how quality assessment may be used to mediate interpretation in heterogeneous evidence syntheses rather than determine theme inclusion or exclusion [96]. While some studies scored low on these metrics, this should not necessarily be interpreted as a reflection of poor study design. Rather, it may highlight an imperfect match between the quality appraisal tools and the purpose of some of the included studies.

The substantial heterogeneity across the included studies represents another limitation. The wide variation in study designs, simulation types, and learner populations poses significant challenges for synthesizing the data. Heterogeneity was examined descriptively by comparing study designs, simulation modalities, learner populations, and the implementation and stated purpose of game elements, rather than by analyzing heterogeneity of effect estimates. Additionally, although we collaborated with professional librarians to develop a comprehensive search strategy and conducted a reference and citation search, it is possible that we might have missed relevant studies since several of the game elements identified in this review were not part of the original search vocabulary.

Finally, because this review is based on secondary data, we were limited to what was reported by the study authors. In many cases, the motivations or intentions behind the use of game elements may have existed but were not documented. A more comprehensive understanding could have been achieved through interviews or direct engagement with simulation designers.

### Conclusion

This is the first comprehensive synthesis of what and how game elements are applied within military trauma simulations, providing a structured evidence base for more intentional and theory-informed design of educational technologies used in high-stakes medical training. Unlike previous reviews, it explicitly focuses on the pedagogical purposes of these elements. It offers an overview of the prevalence of game elements in military trauma care education and synthesizes the pedagogical rationales for their use. While some elements, such as narrative, sensation, and time pressure, were often used in a way that mimics the austerity of the military trauma setting, game elements like badges and competition were underrepresented. Across the reviewed studies, game elements were typically not justified in terms of their pedagogical function. When justifications were provided, they were most often linked to environmental fidelity and immersion, followed by intentions to provide adaptive learning and feedback. None of the included studies in this review correlated individual game elements with the reported learning outcomes. These findings highlight the need for more intentional research on gamification design and transparent reporting, in which the educational purpose of each game element is clearly articulated. Future studies should treat gamification as a set of targeted design choices rather than as a single overarching strategy and further explore how its playful and motivational dimensions can be effectively leveraged in military trauma training to support motivation and learning.

## Appendix

Multimedia Appendix 1 PRISMA 2020 checklist.

Multimedia Appendix 2 PRISMA-S checklist.

Multimedia Appendix 3 PRISMA 2020 abstract cheklist.

Multimedia Appendix 4 SWiM Checklist.

Multimedia Appendix 5 Search strategy.

Multimedia Appendix 6 Game elements per study.

Multimedia Appendix 7 Definition of game elements.

Multimedia Appendix 8 Justification of game elements.

## Abbreviations

MERSQI: Medical Education Research Study Quality Instrument
PRISMA: Preferred Reporting Items for Systematic Reviews and Meta-Analyses
SWiM: Synthesis Without Meta-analysis

## Data Availability

The data sets generated during or analyzed during this systematic review are available from the corresponding author on reasonable request.
