# Supplementary material for: Game Elements in Military Trauma Care Education: Systematic Review
Source: JMIR Serious Games. 2026 Mar 17;14:e79163. doi: 10.2196/79163 (PMC13040169; doi:10.2196/79163)
Supplement: Multimedia Appendix 5 [file games_v14i1e79163_app5.pdf]

Documentation of search strategies
University Library search consultation group

The search strategy was re-run on 14 October 2025. Previous searches were conducted on 26 January 2023, 23 May 2023, 2 July 2024 and 6 February 2025.

Databases:

1. Medline (Ovid)
2. IEEE Xplore
3. ERIC
4. Web of Science
5. ACM Digital Library
6. CINAHL
7. PubMed

Total number of hits:

- Before deduplication: 1,469
- After deduplication: 852

Comments:

Deduplication based on the method described in:
Bramer, W. M., Giustini, D., de Jonge, G. B., Holland, L., & Bekhuis, T. (2016). De-duplication of database search results for systematic reviews in EndNote. *Journal of the Medical Library Association: JMLA*, 104(3), 240–243. doi:10.3163/1536-5050.104.3.014

One final, extra step was added to compare DOI.

1. Medline

Interface: Ovid MEDLINE(R) ALL<br><br>Date of Search: 14 October 2025<br><br>Number of hits: 475<br><br>Comment: In Ovid, two or more words are automatically searched as phrases; i.e. no quotation marks are needed	Field labels<br><br>• exp/ = exploded MeSH term<br>• / = non exploded MeSH term<br>• .ti,ab,kf. = title, abstract and author keywords<br>• adjx = within x words, regardless of order<br>• * = truncation of word for alternate endings<br><br>*added Gamification (new terms since 2022) as MeSH-term

Database(s): **Ovid MEDLINE(R) ALL** 1946 to 14 October 2025
Search Strategy:

#	Searches	Results
1	Computer Simulation/	220396
2	exp Augmented Reality/	1783
3	exp Virtual Reality/	7971
4	exp Ambient Intelligence/	58
5	exp Computer-Assisted Instruction/	12835
6	exp Video Games/	8173
7	exp Simulation Training/	13079
8	Gamification/	140
9	(gamification* or game* or gaming or virtual reality or virtual patient* or virtual environment or virtual patient* or augmented reality or augmented virtuality or mixed reality or merged reality or full immersion).ab,kf,ti.	117392
10	1 or 2 or 3 or 4 or 5 or 6 or 7 or 8 or 9	356530
11	exp Emergency Medicine/ or exp Disaster Medicine/	17131
12	exp Emergency Responders/	16684
13	exp Emergency Nursing/	7638
14	exp Emergency Treatment/	140548
15	exp Trauma Centers/	14228
16	exp "Wounds and Injuries"/	1054434
17	(trauma care or trauma management or bleeding or gun shot* or gunshot* or blast injur* or wound* or surgery or CPR or resuscitation or first aid or first responder or paramedic* or accident* or emergency medicine).ab,kf,ti.	2310430
18	11 or 12 or 13 or 14 or 15 or 16 or 17	3231505
19	exp Military Medicine/	30378

20	exp Military Nursing/	2307
21	(defence medicine* or defense medicine* or combat or war or tactical or military).ti,ab,kf.	170967
22	19 or 20 or 21	185377
23	10 and 18 and 22	475

2. IEEE Xplore

Interface: https://ieeexplore.ieee.org/<br><br>Date of Search: 14 October 2025<br><br>Number of hits: 133	Field labels: All Metadata includes the abstract, index terms, and bibliographic citation data (such as document title, publication title, author, etc.).
("All Metadata":gamification* OR "All Metadata":game* OR "All Metadata":gaming OR "All Metadata":"virtual reality" OR "All Metadata":"virtual environment" OR "All Metadata":"virtual patient" OR "All Metadata":"virtual patients" OR "All Metadata":"augmented reality" OR "All Metadata":"augmented virtuality" OR "All Metadata":"mixed reality" OR "All Metadata":"merged reality" OR "All Metadata":"full immersion")<br><br>AND<br><br>("All Metadata":"trauma care" OR "All Metadata":"trauma management" OR "All Metadata":bleeding OR "All Metadata":"gun shot*" OR "All Metadata":gunshot* OR "All Metadata":"blast injur*" OR "All Metadata":wound* OR "All Metadata":surgery OR "All Metadata":CPR OR "All Metadata":resuscitation OR "All Metadata":"first aid" OR "All Metadata":"first responder" OR "All Metadata":paramedic* OR "All Metadata":accident* OR "All Metadata":"emergency medicine")<br><br>AND<br><br>("All Metadata":"defence medicine" OR "All Metadata":"defence medicines" OR "All Metadata":"defense medicine" OR "All Metadata":"defense medicines" OR "All Metadata":combat OR "All Metadata":war OR "All Metadata":tactical OR "All Metadata":military)	

3. ERIC

Interface: ProQuest<br><br>Date of Search: 14 October 2025<br><br>Number of hits: 8	Field labels: Search performed in all fields
(gamification* or game* or gaming or "virtual reality" or "virtual patient*" or "virtual environment" or "virtual patient*" or "augmented reality" or "augmented virtuality" or "mixed reality" or "merged reality" or "full immersion")<br><br>AND<br><br>("trauma care" or "trauma management" or bleeding or "gun shot*" or gunshot* or "blast injur*" or wound* or surgery or CPR or resuscitation or "first aid" or "first responder" or paramedic* or accident* or "emergency medicine")<br><br>AND<br><br>("defence medicine*" or "defense medicine*" or combat or war or tactical or military)	

4. Web of Science Core Collection

Interface: Clarivate Analytics<br><br>Date of Search: 14 October 2025<br><br>Number of hits: 240	Field labels<br><br>• TS/Topic = title, abstract, author keywords and Keywords Plus<br>• NEAR/x = within x words, regardless of order<br>• * = truncation of word for alternate endings<br><br>Note: sometimes “quotation marks” are needed for single search terms to avoid automatic term mapping (lemmatization).

gamification* or game* or gaming or "virtual reality" or "virtual patient*" or "virtual environment" or "virtual patient*" or "augmented reality" or "augmented virtuality" or "mixed reality" or "merged reality" or "full immersion" (Topic)

AND

"trauma care" or "trauma management" or bleeding or "gun shot*" or gunshot* or "blast injur*" or wound* or surgery or CPR or resuscitation or "first aid" or "first responder" or paramedic* or accident* or "emergency medicine" (Topic)

AND

"defence medicine*" or "defense medicine*" or combat or war or tactical or military (Topic)

5. ACM Digital Library

Interface: ACM Digital Library<br><br>Date of Search: 14 October 2025<br><br>Number of hits: 7	Notes: Searches were performed or different fields sequentially

[[Title: gamification*] OR [Title: game*] OR [Title: gaming] OR [Title: "virtual reality"] OR [Title: "virtual patient*"] OR [Title: "virtual environment"] OR [Title: "virtual patient*"] OR [Title: "augmented reality"] OR [Title: "augmented virtuality"] OR [Title: "mixed reality"] OR [Title: "merged reality"] OR [Title: "full immersion"]] AND [[Title: "trauma care"] OR [Title: "trauma management"] OR [Title: bleeding] OR [Title: "gun shot*"] OR [Title: gunshot*] OR [Title: "blast injur*"] OR [Title: wound*] OR [Title: surgery] OR [Title: cpr] OR [Title: resuscitation] OR [Title: "first aid"] OR [Title: "first responder"] OR [Title: paramedic*] OR [Title: accident*] OR [Title: "emergency medicine"]] AND [[Title: "defence medicine*"] OR [Title: "defense medicine*"] OR [Title: combat] OR [Title: war] OR [Title: tactical] OR [Title: military]]

0 st

[[Abstract: gamification*] OR [Abstract: game*] OR [Abstract: gaming] OR [Abstract: "virtual reality"] OR [Abstract: "virtual patient*"] OR [Abstract: "virtual environment"] OR [Abstract: "virtual patient*"] OR [Abstract: "augmented reality"] OR [Abstract: "augmented virtuality"] OR [Abstract: "mixed reality"] OR [Abstract: "merged reality"] OR [Abstract: "full immersion"]] AND [[Abstract: "trauma care"] OR [Abstract: "trauma management"] OR [Abstract: bleeding] OR [Abstract: "gun shot*"] OR [Abstract: gunshot*] OR [Abstract: "blast injur*"] OR [Abstract: wound*] OR [Abstract: surgery] OR [Abstract: cpr] OR [Abstract: resuscitation] OR [Abstract: "first aid"] OR [Abstract: "first responder"] OR [Abstract: paramedic*] OR [Abstract: accident*] OR [Abstract: "emergency medicine"]] AND [[Abstract: "defence medicine*"] OR [Abstract: "defense medicine*"] OR [Abstract: combat] OR [Abstract: war] OR [Abstract: tactical] OR [Abstract: military]]

7 st

6. Cinahl

Interface: Ebsco<br><br>Date of Search: 14 October 2025<br><br>Number of hits: 149	Field labels<br><br>• MH+ = exploded Cinahl Heading<br>• MH = non exploded Cinahl Heading<br>• TI = title<br>• AB = abstract<br>• Nx = within x words, regardless of order<br>• * = truncation of word for alternate endings

#	Query	Results
S26	S10 AND S19 AND S25	149
S25	S20 OR S21 OR S24	52,533
S24	S22 OR S23	48,336
S23	AB (defence medicine* or defense medicine* or combat or war or tactical or military)	35,039
S22	TI (defence medicine* or defense medicine* or combat or war or tactical or military)	23,062
S21	(MH "Military Nursing")	3,439
S20	(MH "Military Medicine")	4,835
S19	S11 OR S12 OR S13 OR S14 OR S15 OR S18	839,794
S18	S16 OR S17	509,924
S17	AB (trauma care or trauma management or bleeding or gun shot* or gunshot* or blast injur* or wound* or surgery or CPR or resuscitation or first aid or first responder or paramedic* or accident* or emergency medicine or emergency responders or disaster medicine)	398,392
S16	TI (trauma care or trauma management or bleeding or gun shot* or gunshot* or blast injur* or wound* or surgery or CPR or resuscitation or first aid or first responder or paramedic* or accident* or emergency medicine or emergency responders or disaster medicine)	201,615
S15	(MH "Wounds and Injuries+")	339,340
S14	(MH "Trauma Centers")	7,796
S13	(MH "Emergency Treatment+")	68,082
S12	(MH "Emergency Nursing+")	15,726
S11	(MH "Emergency Medicine")	11,936
S10	S1 OR S2 OR S3 OR S4 OR S5 OR S6 OR S9	75,426
S9	S7 OR S8	45,483
S8	AB ("ambient intelligence" or gamification* or game* or gaming or virtual reality or virtual patient* or virtual environment or virtual patient* or augmented reality or augmented virtuality or mixed reality or merged reality or full immersion or "simulation training")	32,648
S7	TI ("ambient intelligence" or gamification* or game* or gaming or virtual reality or virtual patient* or virtual environment or virtual patient* or augmented reality or augmented virtuality or mixed reality or merged reality or full immersion or "simulation training")	22.739
S6	(MH "Gamification")	544
S5	(MH "Video Games+")	6,376
S4	(MH "Computer-Assisted Instruction")	8,356

S3	(MH "Virtual Reality+")	7,144
S2	(MH "Augmented Reality")	825
S1	(MH "Computer Simulation")	19,782

7. PubMed

Interface: PubMed<br><br>Date of Search: 14 October 2025<br><br>Number of hits: 457	Field labels<br><br>• Mesh = exploded MeSH Term<br>• Mesh:NoExp = non exploded MeSH Term<br>• tiab= title/abstract<br>• * = truncation of word for alternate endings

1	"Computer Simulation"[Mesh:NoExp]	220,383
2	"Augmented Reality"[Mesh]	1,780
3	"Virtual Reality"[Mesh]	7,956
4	"Ambient Intelligence"[Mesh]	57
5	"Computer-Assisted Instruction"[Mesh]	12,832
6	"Video Games"[Mesh]	8,171
7	"Simulation Training"[Mesh]	13,061
8	"Gamification"[Mesh:noexp]	140
9	(gamification*[tiab] OR game*[tiab] OR gaming[tiab] OR "virtual reality"[tiab] OR "virtual patient*"[tiab] OR "virtual environment"[tiab] OR "virtual patient*"[tiab] OR "augmented reality"[tiab] OR "augmented virtuality"[tiab] OR "mixed reality"[tiab] OR "merged reality"[tiab] OR "full immersion"[tiab])	117,492
10	#1 OR #2 OR #3 OR #4 OR #5 OR #6 OR #7 OR #8 OR #9	356,589
11	"Emergency Medicine"[Mesh] OR "Disaster Medicine"[Mesh]	17,115
12	"Emergency Responders"[Mesh]	16,670
13	"Emergency Nursing"[Mesh]	7,646
14	"Emergency Treatment"[Mesh]	140,508
15	"Trauma Centers"[Mesh]	14,233
16	"Wounds and Injuries"[Mesh]	1,054,310
17	("trauma care"[tiab] OR "trauma management"[tiab] OR bleeding[tiab] OR "gun shot*"[tiab] OR gunshot*[tiab] OR "blast injur*"[tiab] OR wound*[tiab] OR surgery[tiab] OR CPR[tiab] OR resuscitation[tiab] OR "first aid"[tiab] OR "first responder"[tiab] OR paramedic*[tiab] OR accident*[tiab] OR "emergency medicine"[tiab])	2,309,395
18	#11 OR #12 OR #13 OR #14 OR #15 OR #16 OR #17	3,230,281
19	"Military Medicine"[Mesh]	30,378
20	"Military Nursing"[Mesh]	2,307
21	("defence medicine*"[tiab] OR "defense medicine*"[tiab] OR combat[tiab] OR war[tiab] OR tactical[tiab] OR military[tiab])	164,053
22	#19 OR #20 OR #21	178,439
23	#10 AND #18 AND #22	457
