# Supplementary material for: Game Elements in Military Trauma Care Education: Systematic Review
Source: JMIR Serious Games. 2026 Mar 17;14:e79163. doi: 10.2196/79163 (PMC13040169; doi:10.2196/79163)
Supplement: Multimedia Appendix 6 [file games_v14i1e79163_app6.pdf]

Study	Narrative	Scoring	Badges	Progress	Performance Tables	Content Unlocking	Hints	Challenge	Avatars	Imposed choice	Sensation	Randomness	Difficulty adaptation	Competition	Collaboration	Time pressure
Achatz et al (2020) [40]	x	x		x		x		x	x	x		x	x			x
Arora et al (2014) [41]	x	x						x			x					
Badler et al (1996) [42]	x	x					x	x	x		x	x	x		x	x
Baird et al (2020) [43]	x							x	x	x	x	x				
Beaven et al (2021) [44]	x						x	x			x					
Brown et al (2016) [45]	x	x			x	x	x	x	x	x	x	X	x		x	x
Chi et al (1996) [46]	x	x							x	x	x					
Chi et al (1997) [47]	x	x							x	x	x					

Cohen et al (2013) [48]	x						x	x	x	x	x					x
Couperus et al (2019) [49]	x						x				x					
Couperus et al (2020) [50]	x	x					x			x	x	x	x		x	x
DeFalco et al (2017) [13]	x	x					x	x		x	x		x			x
deLesquen et al (2022) [51]	x	x		x	x				x		x	x	x			x
deLesquen et al (2023) [52]	x	x			x				x		x	x				x
Du et al (2022) [53]	x	x							x	x	x				x	

Freeman et al (2001) [54]	x							x	x	x	x		x			x
Goolsby et al (2014) [55]	x						x				x					x
Hemman et al (2005) [56]	x						x									
Henderson et al (1986) [57]	x	x					x	x		x	x	x	x	x	x	x
Henderson et al (2020) [58]	x							x		x	x		x			
Kyle et al (2004) [59]	x						x	x			x					x
Lombardo et al (2022) [60]	x	x								x	x				x	

Lu et al (2023) [61]	x	x								x	x					
Lennquist Montán et al (2014) [62]	x	x									x	x	x			x
Netzer et al (2015) [63]	x	x						x					x			x
Pasquier et al (2016) [11]	x	x	x		x	x	x	x		x	x		x	x		x
Planchon et al (2018) [12]	x	x								x	x					
Qin et al (2024) [64]	x	x			x		x			x	x	x				x
Rabotin et al (2024) [65]	x	x			x					x	x		x			x
Satava and Jones (1996) [66]	x						x	x	x		x					

Sonesson et al (2023) [67]	x									x			x			
Sotomayor et al (2008) [68]	x						x	x		x	x					
Sotomayor et al (2010) [14]	x										x					
Stansfield et al (1998) [69]	x	x					x	x	x	x	x					x
Stathakarou et al (2024) [30]	x	x				x	x	x		x	x		x			x
Stone et al (2005) [70]	x	x			x		x			x	x					x
Stone et al (2011) [71]	x									x	x					x
Stone et al (2017) [72]	x							x			x				x	x

Tretyak et al (2025) [73]	x							x	x		x					x
Wier et al (2017) [74]	x							x			x					x
Willy et al (1998) [75]	x						x			x	x		x			x
Zhu et al (2024) [76]	x	x		x	x		x			x	x					x
Total number	42	24	1	3	8	4	20	21	14	27	37	10	16	2	7	26
