# Supplementary material for: Game Elements in Military Trauma Care Education: Systematic Review
Source: JMIR Serious Games. 2026 Mar 17;14:e79163. doi: 10.2196/79163 (PMC13040169; doi:10.2196/79163)
Supplement: Multimedia Appendix 7 [file games_v14i1e79163_app7.pdf]

Game elements	Definition
Narrative	The structured sequence of events and choices that shape the learner’s experience.
Sensation	The use of sensory stimuli, such as visual and auditory cues, to enhance immersion.
Imposed choice	Providing learners with multiple decision options and requiring them to select a specific one to proceed, preventing further progress in the virtual patient scenario until the correct choice is made.
Time pressure	Requiring learners to perform actions or make decisions under time constraints
Scoring	Assigning points for correct actions or decisions and deducting points for incorrect ones to provide feedback and encourage improvement.
Hints	Clues provided to help learners find the correct answers without giving them away directly.
Challenge	Elements designed to test the learner’s abilities and maintain engagement.
Difficulty adaptation	Adjusting the level of difficulty based on the learner’s performance.
Avatars	Digital representations of the learners or patients within the virtual patient environment.
Randomness	Introducing unpredictable elements into the simulation to create a more realistic and challenging environment.
Performance tables	Detailed overviews of the learner’s performance across multiple tasks or criteria.
Collaboration	Game mechanics that require or encourage learners to interact and work with others to achieve shared goals or complete tasks.
Content unlocking	Access to new content or pathways based on the learner’s performance or decisions.
Progression	Tracking and visualizing a learner’s development and achievements over time.
Competition	Encouraging learners to compete with each other, often through leaderboards or similar mechanisms.
Badges	Visual symbols of achievement awarded for completing specific tasks or reaching milestones.
