# Supplementary material for: Game Elements in Military Trauma Care Education: Systematic Review
Source: JMIR Serious Games. 2026 Mar 17;14:e79163. doi: 10.2196/79163 (PMC13040169; doi:10.2196/79163)
Supplement: Multimedia Appendix 8 [file games_v14i1e79163_app8.pdf]

Study	Quotes	Theme
Achatz et al (2020) [40]	Time pressure: "Real-time conditions are simulated with the help of a timescale"	Realism and emotional engagement
Arora et al (2014) [41]	Narrative & Sensation: The simulation includes "ambient noise and temperatures, hospital layout, clinical, administration and discharge processes, complex patient cases, equipment, and full transport support including helicopter patient transfer… recreates the hospital and its processes in its entirety"… "For truly contextualized learning to occur, a simulation should mirror the clinical environment as closely as possible by providing an opportunity that would allow multiple clinicians to interact in real time across a fully integrated care pathway."	Realism and emotional engagement
Baird et al (2020) [43]	Narrative & Sensation: "This module, and all future modules, has been shaped by USAISR subject matter experts (SMEs) to incorporate relevant detail, common pitfalls, and as much realism as possible. The scenario presented to the user upon patient arrival is a military Service Member (SM) covered with dirt and blood. It is verbally communicated that the patient was in a dismounted improvised explosive device (IED) blast and has suffered severe burn wounds as a result."	Realism and emotional engagement
Brown et al (2016) [45]	Challenge: "These variables take the form of sandstorms, vehicles passing by, and helicopters passing over. The variables serve to add auditory and visual distractions, adding to the user's overall cognitive load, especially when attempting to coordinate verbally with other users."<br><br>Narrative, Sensation, Randomization, Avatar: "To further immerse the player within the virtual environment, immersion variables function to add life and variation." "To imbue the experience of realistic battlefield casualty… the user's perspective and experience mirrors true to- life conditions: performing treatment on the casualty in a	Realism and emotional engagement<br><br>Realism and emotional engagement

	crouched position, viewing the world from a first-person perspective with only the character's own hands visible and the ability to move and look freely around the virtual environment."<br><br>Difficulty adaptation: "Since each user may have a varying amount of treatment knowledge and experience, a difficulty system is implemented that allows users to select a difficulty level for any of the three training scenarios. The difficulty system allows for progressively challenging game conditions that best suit the skill level of the user."<br><br>Performance tables: "Upon conclusion of casualty treatment, the user is presented with detailed performance feedback via an interface with an overview of each key user action and event. This overview presents potential improvement areas for self directed individual and team players."	 <br>Adaptive learning and feedback<br><br>Adaptive learning and feedback
Chi et al (1996) [46]	Sensation: "A casualty's state is then linked with various visual effects to imitate the appearance and behavior of real Casualties... Concurrent research is being done to create wound appearances and motor behavior with the goal of simulating casualties who are realistic enough to elicit appropriate diagnostic and therapeutic behavior from the medical corpsman trainee."	Realism and emotional engagement
Chi et al (1997) [47]	Scoring: "During the training process, it can aid the trainee in associating physiological states and consequences with the performance (or omission) of specific medical procedures."<br><br>Imposed choice: "The command list allows the trainee to ask the casualty questions to assess the situation as in real emergency medical situations"	Adaptive learning and feedback<br><br>Realism and emotional engagement

DeFalco et al (2017) [13]	Challenge: "It was anticipated that laying out the scenarios in this manner would increase the frequency of frustration among the participants. This increase of frustration would be a result of the fact that while the first scenario would lead participants to believe that this was a game that was winnable, the second no-win Kobayashi Maru scenario would undermine those beliefs."<br><br>Imposed choice: "Problem scenarios in TC3Sim are structured linearly, presenting a fixed series of events regardless of the learner's actions. However, learners can freely navigate the 3D virtual environments in TC3Sim, as well as choose how to administer casualty care under fire. While TC3Sim supports a considerable amount of learner agency, its training scenarios are designed to direct players towards the game's objectives (e.g., administering care), guiding the learner toward key learning objectives."	Frustration<br><br>Learner agency
de Lesquen et al (2022) [51]	Narrative & Sensation: "Spatial immersion: The trainee has to feel a perceptually convincing simulated world. The CCP should take place in a neutral place, such as a sports hall, an industrial building, or a warehouse"<br><br>Avatars: "Creating engaging avatars is crucial for social immersive experiences to catch on with trainees."<br><br>Difficulty adaptation: "the Traumasims conception validates the main features of high-fidelity medical simulation known to lead to effective learning: the repetition of scenarii, providing feedback at the end of each scenario, graduate degrees of difficulty by varying numbers and levels of severity of the victims in each scenario, the capture of clinical variation in a controlled VE, and individualized learning"<br><br>Scoring & performance tables: "At the end of the scenario, brief and concise personnel feedback was delivered to the trainee as an essential component of in situ training. Medusims designed a correcting grid for scoring and	Realism and emotional engagement<br><br>Realism and emotional engagement<br><br>Adaptive learning and feedback<br><br>Adaptive learning and feedback

	personalized debriefing. The scoring system integrates the time to evacuation, categorization, life-saving actions, and teamwork ability.”	
Du et al (2022) [53]	Collaboration: “A multiplayer design within the XR environment is thought to be able to offer a chance to rotate through different team roles, supporting an active and immersive learning experience with the potential to equip the learners with the crucial teamwork skills required for medical care”	Realism and emotional engagement
Freeman et al (2001) [54]	Difficulty adaptation: “We selected three specific cases to demonstrate important teaching points and to progressively challenge the trainee.”<br><br>Narrative & Sensation: “In this project, collaborators adapted and merged existing technologies to produce a flexible, modular patient simulation system with both three-dimensional virtual reality and two dimensional flat screen user interfaces for teaching cognitive assessment and treatment skills. This experiential, problem-based training approach engages the user in a stress-filled, high fidelity world, providing multiple learning opportunities within a compressed period of time and without risk.”	Challenge the learners<br><br>Risk-free, experiential learning
Henderson et al (1986) [57]	Narrative, Sensation, Randomness: “The program stresses realism: military, clinical, and interpersonal. The inherent drama of wartime medicine and casualty care is exploited to involve the student and draw him in a personal way into the situations presented. As in real life, the student cannot predict in advance what case he will see, even if he has used the program before. Also, doing the same thing in apparently similar situations will not always have the intended result.”	Realism and emotional engagement
Lombardo et al (2022) [60]	Scoring: “We collaboratively developed a virtual reality simulator that offers immediate feedback through real-time patient physiologic responses and overall grading in training scenarios without requiring an instructor or facilitator”	Adaptive learning and feedback

Lennquist Montán et al (2014) [62]	Imposed choice: "To the card could also be attached movable treatment tags indicating performed treatments (Fig. 2). For prehospital management, 18 such different treatment alternatives were available and for in-hospital management, an additional 23 were available, in proportions and numbers adjusted to the size of the hospital. Every treatment was associated with a time, based on the results from time studies of medical staff carrying out the same procedures. The access to tags for the trainee could be adjusted to the access in reality."	Realism and emotional engagement
Netzer et al (2015) [63]	Narrative & sensation: "Training was performed onboard a military naval vessel, in maximally realistic conditions, thus rehearsing a scenario of care in the genuine theater of operation. Medical immersion training is emerging as a teaching standard."	Realism and emotional engagement
Pasquier et al (2016) [11]	Competition & Scoring: "Furthermore, through the processes of scoring and gamification applied in 3D-SC1, the trainee is motivated to improve his personal experience. He also shares his scores with his peers in a competitive and engaging challenge"<br><br>Scoring & Performance tables: "Most importantly, at the end of the 3D-SC1 simulation, a personalized debriefing is proposed (Figure 5), highlighting good performance achieved in the experience, the procedures for which the trainee has to improve, and the missed procedures for which the automatic virtual instruc- tor had to take the control of the experience to perform the procedure".<br><br>Sensation: "A common criticism about SGs is that the dynamic colorful world of a computer game will distract the trainee's attention from the learning process. However, current soldiers grew up with digital media and have developed a much better aptitude to relate a virtual world to reality."	Motivation, engagement<br><br>Adaptive learning and feedback<br><br>Realism and emotional engagement

Rabotin et al (2023) [65]	Difficulty adaptation: "Adaptive learning – utilizing the individual and aggregative performance data as the substrate for designing consequent training sessions, thus creating each training event as a custom challenge for the trainee, maintaining improvement along the individual learning curve."	Adaptive learning and feedback
Stansfield et al (1998) [69]	Time pressure: "Even if the trainee performs all steps correctly, if they are not done in a timely manner the patient will die. Fortunately, in the case of MediSim, the trainee is permitted to make such a catastrophic error, with its accompanying lessons learned, without causing a real-life fatality."<br><br>Challenge: "The goal of the MediSim trainer, in contrast, is to train rapid situational assessment and decision-making under highly stressful conditions. Thus, MediSim looks to train the medic not to insert an IV, but rather to understand the circumstances under which an IV is required. The former is referred to as task training, while the latter is referred to as situational training."	Risk-free experiential learning<br><br>Situational awareness
Stathakarou et al (2024) [30]	Narrative, Sensation, Time-pressure: "key design principle was to "emphasize a feeling of presence by keeping the patient visible." We aimed to emphasize the participants' control and the feeling of being in charge rather than a spectator. The interaction with the VP focused on the user's next step, enhancing emotional involvement and placing the responsibility for patient care on the learners, requiring them to act quickly and manage the visible trauma."<br><br>Imposed choice: "VPs were designed to prompt learners to make decisions as if they were a military medic using first-person language, for example, "I decide to approach the injured soldier." This approach aimed to immerse the learner in the role and responsibility of a military medic."	Realism and emotional engagement<br><br>Realism and emotional engagement

Tretyak et al (2025) [73]	Challenge, Time pressure: ''To enhance realism and decision-making under pressure, the environment includes time constraints and distracting stimuli. ''	Realism and emotional engagement
Wier et al (2017) [74]	Challenge, sensation: ''Smoke generators and debris scattered across the floor can add to the realism of the training environment, as can the simulation of flying bullets, screams, and explosions. These simulations may feel "extreme" to the student because of sources of additional stress. The presence of a high-stress simulated environment may negatively impact student decision-making ability, team communication, and performance.22–24 However, exposing students to this type of stress inoculation training may allow them to overcome anxiety and reduce emotional reaction or shock in operational environments''	Emotional regulation and overcoming anxiety
Willy et al (1998) [75]	Imposed choice: ''The ability to interactively retrieve additional patient data makes the computer simulation even more realistic''	Realism and emotional engagement
